# Effectiveness of COVID-19 mRNA Vaccination in Preventing COVID-19–Associated Hospitalization Among Adults with Previous SARS-CoV-2 Infection — United States, June 2021–February 2022

**DOI:** 10.15585/mmwr.mm7115e2

**Published:** 2022-04-15

**Authors:** Ian D. Plumb, Leora R. Feldstein, Eric Barkley, Alexander B. Posner, Howard S. Bregman, Melissa Briggs Hagen, Jacqueline L. Gerhart

**Affiliations:** ^1^Division of Viral Diseases, National Center for Immunization and Respiratory Diseases, CDC; ^2^CDC COVID-19 Emergency Response Team; ^3^Epic Research, Epic Systems Corporation, Verona, Wisconsin.

Previous infection with SARS-CoV-2, the virus that causes COVID-19, has been estimated to confer up to 90% protection against reinfection, although this protection was lower against the Omicron variant compared with that against other SARS-CoV-2 variants (*1*–*3*). A test-negative design was used to estimate effectiveness of COVID-19 mRNA vaccines in preventing subsequent COVID-19–associated hospitalization among adults aged ≥18 years with a previous positive nucleic acid amplification test (NAAT) or diagnosis of COVID-19.^†^ The analysis used data from Cosmos, an electronic health record (EHR)–aggregated data set (*4*), and compared vaccination status of 3,761 case-patients (positive NAAT result associated with hospitalization) with 7,522 matched control-patients (negative NAAT result). After previous SARS-CoV-2 infection, estimated vaccine effectiveness (VE) against COVID-19–associated hospitalization was 47.5% (95% CI = 38.8%–54.9%) after 2 vaccine doses and 57.8% (95% CI = 32.1%–73.8%) after a booster dose during the Delta-predominant period (June 20–December 18, 2021), and 34.6% (95% CI = 25.5%–42.5%) after 2 doses and 67.6% (95% CI = 61.4%–72.8%) after a booster dose during the Omicron-predominant period (December 19, 2021–February 24, 2022). Vaccination provides protection against COVID-19–associated hospitalization among adults with previous SARS-CoV-2 infection, with the highest level of protection conferred by a booster dose. All eligible persons, including those with previous SARS-CoV-2 infection, should stay up to date with vaccination to prevent COVID-19–associated hospitalization.

Data were obtained from Cosmos (*4*), an EHR data set that includes information from more than 135 million patients and 154 health care organizations in the United States.^§^ Patients eligible for inclusion in the analysis met the following four criteria: 1) age ≥18 years, 2) residence in the United States, 3) at least one hospital admission for a COVID-19–like illness,^¶^ with a hospitalization-associated NAAT performed from 10 days before through 3 days after admission during June 20, 2021–February 24, 2022, and 4) a previous positive NAAT result or diagnostic code of COVID-19 (with or without hospitalization) >90 days before the date of the NAAT associated with the subsequent hospitalization.** Patients under the billing category of “observation” and patients who were admitted and discharged on the same day were excluded. Vaccination status was categorized on the day of the NAAT associated with the hospitalization as 1) unvaccinated, 2) after dose 1, 3) after dose 2, or 4) after a booster dose^††^; patients were excluded if they did not meet one of these definitions or if the previous positive NAAT result or COVID-19 diagnosis was after the date of the most recent vaccine dose. Vaccination information was collected during the 14 days after hospitalization or other health care visit from a patient’s health system, other health systems via clinical record exchanges, state registries, and patient-reported history.^§§^

VE was estimated using conditional logistic regression, comparing the vaccination status among case-patients and control-patients. VE after each vaccine dose was estimated using the unvaccinated group as a referent. For estimation of relative VE after a booster dose, the referent group had received dose 2 (but not a booster dose) ≥5 months previously. Eligible case-patients were matched with control-patients using a 1:2 ratio by 2-week period of the hospitalization-associated NAAT, 10-year age group, and state of residence. After matching, estimates were adjusted for sex, race/ethnicity, number of clinical encounters during 2019, number of underlying health conditions, and days since the previous infection.^¶¶^ The period June 20–December 18, 2021, was categorized as Delta-predominant, and the period December 19, 2021–February 24, 2022, as Omicron-predominant; periods were defined as range of dates when estimated national prevalence of a SARS-CoV-2 variant exceeded 50%.*** In a sensitivity analysis, VE was also estimated defining previous infection as a positive NAAT result. Wilcoxon rank-sum tests and chi-square tests were used to compare group medians and proportions, respectively; p-values <0.05 were considered statistically significant. Data were analyzed using R software (version 4.1.2; R Foundation). This activity was reviewed by CDC and was conducted consistent with applicable federal law and CDC policy.^†††^

Among 5,116,024 adults aged ≥18 years with an initial positive NAAT result or diagnosis of COVID-19, 51,609 patients were hospitalized with COVID-19–like illness associated with a NAAT result >90 days after the previous infection,^§§§^ including 5,048 (9.8%) with a positive NAAT result. Among these 5,048 case-patients, 2,436 (48.3%; median = 67 reinfections per week) were admitted during the Delta-predominant period, and 2,612 (51.7%; median = 343 reinfections per week) during the Omicron-predominant period (Supplementary Figure, https://stacks.cdc.gov/view/cdc/116026).

After 7,569 patients were excluded, 11,283 of 44,040 eligible patients were matched and included in the analysis, 3,761 (87.1%) of 4,319 eligible case-patients and 7,522 (18.9%) of 39,721 eligible control-patients. Case- and control-patients were demographically similar, with fewer underlying conditions and previous health care encounters among case-patients (Table 1). Overall, 61.2% of case-patients were unvaccinated, 4.3% had received 1 vaccine dose, 27.6% had received 2 doses, and 6.9% had received a booster dose, compared with 47.5%, 5.5%, 33.2%, and 13.9% of control-patients, respectively.

**TABLE 1:** Characteristics of hospitalized adults with previous SARS-CoV-2 infection,* by subsequent nucleic acid amplification test result^†^— United States, June 2021–February 2022^§^

Characteristic	No. (column %)			p-value**
	Total (N = 11,283)	Case-patients (NAAT-positive)^¶^ (n = 3,761)	Control-patients (NAAT-negative)^¶^ (n = 7,522)	
**Age group, yrs**				
18–29	993 (8.8)	331 (8.8)	662 (8.8)	>0.990
30–44	1,717 (15.2)	573 (15.2)	1,144 (15.2)	
45–64	3,804 (33.7)	1,273 (33.8)	2,531 (33.6)	
≥65	4,769 (42.3)	1,584 (42.1)	3,185 (42.3)	
**Sex**				
Women	6,391 (56.6)	2,114 (56.2)	4,277 (56.9)	0.510
Men	4,892 (43.4)	1,647 (43.8)	3,245 (43.1)	
**Race and ethnicity**				
White, non-Hispanic	6,963 (61.7)	2,286 (60.8)	4,677 (62.2)	0.026
Black, non-Hispanic	2,821 (25.0)	924 (24.6)	1,897 (25.2)	
Hispanic	1,131 (10.0)	413 (11.0)	718 (9.5)	
Other, non-Hispanic^††^	368 (3.3)	138 (3.7)	230 (3.1)	
**Underlying health conditions^§§^**				
0	536 (4.8)	198 (5.3)	338 (4.5)	<0.001
1	1,610 (14.3)	641 (17.0)	969 (12.9)	
>1	9,137 (81.0)	2,922 (77.7)	6,215 (82.6)	
**Vaccination status** ^¶¶^				
Unvaccinated	5,874 (52.1)	2,303 (61.2)	3,571 (47.5)	<0.001
Any mRNA vaccine, 1 dose	574 (5.1)	161 (4.3)	413 (5.5)	
Any mRNA vaccine, 2 doses	3,534 (31.3)	1,038 (27.6)	2,496 (33.2)	
Any mRNA vaccine, booster dose	1,301 (11.5)	259 (6.9)	1,042 (13.9)	
**Clinical encounters during 2019**				
0	2,403 (21.3)	781 (20.8)	1,622 (21.6)	<0.001
1–9	4,199 (37.2)	1,628 (43.3)	2,571 (34.2)	
≥10	4,681 (41.5)	1,352 (35.9)	3,329 (44.3)	
**Month of hospital admission**				
Jun 2021	156 (1.4)	54 (1.4)	102 (1.4)	0.930
Jul 2021	528 (4.7)	179 (4.8)	349 (4.6)	
Aug 2021	982 (8.7)	320 (8.5)	662 (8.8)	
Sep 2021	874 (7.7)	294 (7.8)	580 (7.7)	
Oct 2021	621 (5.5)	204 (5.4)	417 (5.5)	
Nov 2021	583 (5.2)	198 (5.3)	385 (5.1)	
Dec 2021	1,875 (16.6)	601 (16.0)	1,274 (16.9)	
Jan 2022	4,555 (40.4)	1,548 (41.2)	3,007 (40.0)	
Feb 2022	1,109 (9.8)	363 (9.7)	746 (9.9)	
Hospitalization variant predominance period***				
B.1.617.2 (Delta)	4,385 (38.9)	1,437 (38.2)	2,948 (39.2)	0.310
B.1.1.529 (Omicron)	6,898 (61.1)	2,324 (61.8)	4,574 (60.8)	
**U.S. Census region**				
Northeast	2,340 (20.7)	780 (20.7)	1,560 (20.7)	
Midwest	3,300 (29.2)	1,100 (29.2)	2,200 (29.2)	>0.990
South	5,133 (45.5)	1,711 (45.5)	3,422 (45.5)	
West	510 (4.5)	170 (4.5)	340 (4.5)	
Initial infection variant predominance period***				
Pre-Delta*	9,593 (85.0)	3,226 (85.8)	6,367 (84.6)	0.110
B.1.617.2 (Delta)^5^	1,690 (15.0)	535 (14.2)	1,155 (15.4)	
Initial diagnosis source*				
COVID-19 diagnosis	4,250 (37.7)	1,615 (42.9)	2,635 (35.0)	<0.001
NAAT result	1,013 (9.0)	317 (8.4)	696 (9.3)	
Both	6,020 (53.4)	1,829 (48.6)	4,191 (55.7)	
**Initial infection to NAAT associated with hospitalization, days^†^**				
90–119	735 (6.5)	287 (7.6)	448 (6.0)	<0.001
120–179	1,389 (12.3)	479 (12.7)	910 (12.1)	
180–269	1,787 (15.8)	552 (14.7)	1,235 (16.4)	
270–364	2,402 (21.3)	711 (18.9)	1,691 (22.5)	
≥365	4,970 (44.0)	1,732 (46.1)	3,238 (43.0)	

**Abbreviation:** NAAT = nucleic acid amplification test.

* Initial diagnosis was based on a previous positive SARS-CoV-2 NAAT or clinical diagnosis of COVID-19 >90 days before the date of the NAAT associated with subsequent hospitalization. COVID-19 was defined as a clinical encounter with any of the following *International Classification of Diseases, Tenth Revision* diagnostic codes: U07.1, J12.81, or J12.82.

^†^ Defined as NAAT performed between 10 days before and 3 days after the date of hospital admission with a diagnosis of COVID-19-like illness. COVID-19–like illness diagnoses were defined based on others’ methods (https://www.nejm.org/doi/full/10.1056/nejmoa2110362, Supplement Table S2) and included acute respiratory illness (e.g., COVID-19, respiratory failure, or pneumonia) or related signs or symptoms (e.g., cough, fever, dyspnea, vomiting, or diarrhea) using diagnostic codes from the *International Classification of Diseases, Tenth Revision*.

^§^ Patients were eligible for inclusion if the hospitalization-associated SARS-CoV-2 NAAT was performed during June 20, 2021–February 24, 2022.

^¶^ Cases had a positive SARS-CoV-2 NAAT result associated with hospitalization; controls had a negative SARS-CoV-2 NAAT result associated with hospitalization.

** Wilcoxon rank-sum tests and chi-square tests were used to compare medians and proportions, respectively; p-values <0.05 were considered statistically significant.

^††^ Other non-Hispanic includes Asian, Native Hawaiian or other Pacific Islander, and American Indian or Alaska Native persons.

^§§^ Underlying conditions were extracted from electronic health record clinical encounter data and were based on a CDC list of conditions associated with the highest risk for COVID-19 (https://www.cdc.gov/coronavirus/2019-ncov/hcp/clinical-care/underlyingconditions.html, accessed March 23, 2022), and included the following: alcoholic liver disease, autoimmune hepatitis, bronchiectasis, bronchopulmonary dysplasia, cancer, cardiomyopathy, cerebrovascular disease, chronic kidney disease, cirrhosis, chronic obstructive pulmonary disease, coronary artery disease, current smoker, administration or prescription of nontopical glucocorticoids within the previous 12 months, heart failure, HIV, immune deficiency, administration or prescription of immunosuppressive medications within the previous 12 months, interstitial lung disease, nonalcoholic fatty liver disease, obesity, pulmonary arterial hypertension, pulmonary embolus, pregnancy, solid organ transplant, tuberculosis, and type 1 or 2 diabetes. Among these, diagnoses associated with immunocompromise had overall similar prevalence between cases and controls, including immunosuppressive medications other than steroids (7.9% of case-patients and 7.3% of control-patients), immune deficiencies (4.4% of case-patients and 4.5% of control-patients), solid organ transplant recipients (2.4% of case-patients and 1.8% of control-patients) and HIV (0.9% of case-patients and 0.9% of control-patients).

^¶¶^ Patients were categorized on the date of NAAT associated with hospitalization as unvaccinated if no COVID-19 vaccine had been received; after dose 1 if ≥14 days had elapsed since receipt of the first dose of an mRNA COVID-19 vaccine and before any second dose; after dose 2 if ≥14 days had elapsed since receipt of the second dose of an mRNA COVID-19 vaccine, and no subsequent dose was received; and after a booster dose if ≥14 days had elapsed since receipt of an mRNA booster dose administered ≥5 months after a second dose. Patients were excluded from the analysis if they received a non-mRNA COVID-19 vaccine; the day of the NAAT-associated hospitalization was <14 days after dose 1, dose 2, or a booster dose; dose 2 was received <14 days after dose 1; any booster dose was <5 months after dose 2, they received >3 doses of vaccine, or their previous positive NAAT result or COVID-19 diagnosis was after date of the most recent vaccine dose. Median time from receipt of dose 1 to dose 2 was 21 days (IQR = 21–24) for Pfizer-BioNTech and 28 days (IQR = 28–30) for Moderna vaccines. Median time from receipt of dose 2 to dose 3 was 232 days (IQR = 203–258) for Pfizer-BioNTech and 236 days (IQR = 210–261) for Moderna vaccines.

*** Periods were defined as a range of dates when estimated national prevalence of a SARS-CoV-2 variant exceeded 50% as pre-Delta (before June 20, 2021), Delta (during June 20, 2021–December 18, 2021), and Omicron (from December 19, 2021). https://covid.cdc.gov/covid-data-tracker/#variant-proportions

During the Delta-predominant period, estimated adjusted VE was 58.8% (95% CI = 41.3%–71.1%) after dose 1, 47.5% (95% CI = 38.8%–54.9%) after dose 2, and 57.8% (95% CI = 32.1%–73.8%) after a booster dose; during the Omicron-predominant period, adjusted VE was 33.0% (95% CI = 15.0%–47.2%) after dose 1, 34.6% (95% CI = 25.5%–42.5%) after dose 2, and 67.6% (95% CI = 61.4%–72.8%) after a booster dose (Table 2). VE estimates were similar whether hospitalizations were <90 days or ≥90 days after the most recent vaccine dose. Similar estimates were obtained in a sensitivity analysis that included 2,146 case-patients and 4,887 control-patients with previous infection confirmed by NAAT (Supplementary Table, https://stacks.cdc.gov/view/cdc/116025).

**TABLE 2:** Estimated vaccine effectiveness against hospitalization with COVID-19 after previous SARS-CoV-2 infection* — United States, June 2021–February 2022

Variant period/Vaccination status	No. of case-patients^†^ (N = 3,761)	No. of control-patients^†^ (N = 7,522)	VE^§^ (95% CI)	
			Unadjusted	Adjusted
**Overall**				
Unvaccinated (Ref)	2,303	3,571	—	—
Any mRNA vaccine, 1 dose^¶,^**	161	413	41.6 (29.3–51.8)	41.9 (29.5–52.1)
Any mRNA vaccine, 2 doses^¶,^**	1,038	2,496	38.2 (32.2–43.7)	39.4 (33.3–45.0)
Pfizer-BioNTech^¶^	588	1,432	40.8 (33.1–47.5)	42.7 (35.0–49.4)
Moderna^¶^	450	1,064	37.1 (27.6–45.3)	38.7 (29.1–46.9)
Any mRNA vaccine, booster dose^¶,^**	259	1,042	66.4 (60.7–71.3)	67.0 (61.3–71.9)
**Delta predominant**				
Unvaccinated (Ref)	950	1,468	—	—
Any mRNA vaccine, 1 dose^¶^	45	171	61.0 (44.7–72.5)	58.8 (41.3–71.1)
Any mRNA vaccine, 2 doses^¶^	415	1,209	50.7 (42.9–57.5)	47.5 (38.8–54.9)
Pfizer-BioNTech^¶^	234	678	52.8 (42.8–61.1)	50.0 (39.0–59.0)
Moderna^¶^	181	531	47.9 (35.3–58.1)	44.0 (29.9–55.2)
Any mRNA vaccine, booster dose^¶^	27	100	60.2 (36.4–75.0)	57.8 (32.1–73.8)
**Omicron predominant**				
Unvaccinated (Ref)	1,353	2,103	—	—
Any mRNA vaccine, 1 dose^¶^	116	242	27.3 (8.14–42.5)	33.0 (15.0–47.2)
Any mRNA vaccine, 2 doses^¶^	623	1,287	26.9 (17.4–35.4)	34.6 (25.5–42.5)
Pfizer-BioNTech^¶^	354	754	29.2 (16.9–39.7)	37.3 (25.8–46.9)
Moderna^¶^	269	533	26.2 (10.8–39.0)	35.9 (21.7–47.4)
Any mRNA vaccine, booster dose^¶^	232	942	64.6 (58.1–70.2)	67.6 (61.4–72.8)
**Relative VE of booster dose compared with primary series** ^††^				
**Overall**				
≥5 months after second dose (Ref)^††^	697	1,536	—	—
Any mRNA vaccine, booster dose^††^	259	1,042	56.5 (44.6–65.9)	55.9 (43.6–65.5))

**Abbreviations:** NAAT = nucleic acid amplification test; Ref = referent group; VE = vaccine effectiveness.

* Initial diagnosis was based on a previous positive SARS-CoV-2 NAAT or clinical diagnosis of COVID-19 >90 days before the date of the NAAT associated with subsequent hospitalization. COVID-19 was defined as a clinical encounter with any of the following *International Classification of Diseases, Tenth Revision* diagnostic codes: U07.1, J12.81, or J12.82.

^†^ Case-patients had a positive NAAT performed 10 days before through 3 days after the date of hospitalization with a diagnosis of COVID-19-like illness; control-patients had a negative NAAT result. COVID-19–like illness diagnoses were defined based on other methods (https://www.nejm.org/doi/full/10.1056/nejmoa2110362, Supplement Table S2) and included acute respiratory illness (e.g., COVID-19, respiratory failure, or pneumonia) or related signs or symptoms (e.g., cough, fever, dyspnea, vomiting, or diarrhea) using diagnostic codes from the *International Classification of Diseases, Tenth Revision*. Patients were eligible for inclusion if the hospitalization-associated SARS-CoV-2 NAAT was performed during June 20, 2021–February 24, 2022.

^§^ VE was calculated as [1 − odds ratio] x 100, estimated using conditional logistic regression in a test-negative design after matching by 2-week calendar period of NAAT associated with hospital admission, 10-year age group, and state of residence. Adjusted estimates accounted in addition for measured differences in sex, race/ethnicity (White non-Hispanic race: yes/no and Hispanic ethnicity: yes/no), number of clinical encounters during 2019 (0, 1–9, or ≥10), number of underlying conditions (0, 1, or >1), and days since previous infection (as a continuous variable). Underlying conditions were extracted from EHR clinical encounter data and based on a CDC list of conditions associated with the highest risk for COVID-19 (https://www.cdc.gov/coronavirus/2019-ncov/hcp/clinical-care/underlyingconditions.html, accessed March 23, 2022), including the following diagnoses: alcoholic liver disease, autoimmune hepatitis, bronchiectasis, bronchopulmonary dysplasia, cancer, cardiomyopathy, cerebrovascular disease, chronic kidney disease, cirrhosis, chronic obstructive pulmonary disease, coronary artery disease, current smoker, administration or prescription of nontopical glucocorticoids within the previous 12 months, heart failure, HIV, immune deficiency, administration or prescription of immunosuppressive medications within the previous 12 months, interstitial lung disease, nonalcoholic fatty liver disease, obesity, pulmonary arterial hypertension, pulmonary embolus, pregnancy, solid organ transplant, tuberculosis, and type 1 or 2 diabetes.

^¶^ Patients were categorized on the date of NAAT associated with hospitalization as unvaccinated if no COVID-19 vaccine had been received; after dose 1 if ≥14 days had elapsed since receipt of the first dose of an mRNA COVID-19 vaccine and before any second dose; after dose 2 if ≥14 days had elapsed since receipt of the second dose of an mRNA COVID-19 vaccine and no subsequent dose was received; and after a booster dose if ≥14 days had elapsed since receipt of an mRNA booster dose administered ≥5 months after a second dose. Patients were excluded from the analysis if they received a non-mRNA COVID-19 vaccine; the day of the NAAT-associated hospitalization was <14 days after dose 1, dose 2, or a booster dose; dose 2 was received <14 days after dose 1; any booster dose was <5 months after dose 2, they received >3 doses of vaccine, or the previous positive NAAT result or COVID-19 diagnosis was after the date of the most recent vaccine dose.

** Among persons with a previous infection, adjusted VE <90 days after dose 1 was 42.0% (95% CI = 16.8%–59.5%) and ≥90 days after dose 1 was 42.2% (95% CI = 26.0%–54.8%); adjusted VE <90 days after dose 2 was 44.6% (95% CI = 28.6%–56.9%) and ≥90 days after dose 2 was 39.3% (95% CI = 32.4%–45.4%); and adjusted VE <90 days after dose 3 was 67.9% (95% CI = 60.3%–74.0%) and ≥90 days after dose 3 was 62.4% (95% CI = 48.6%–72.5%).

^††^ For estimation of relative VE after a booster dose, the referent group had received dose 2 (but not a booster dose) ≥5 months previously.

During the analysis period, among persons who had a previous positive NAAT result or COVID-19 diagnosis before the first vaccine dose, estimated VE was 43.1% (95% CI = 30.7%–53.2%) after dose 1, 41.7% (95% CI = 35.5%–47.3%) after dose 2, and 70.3% (95% CI = 64.1%–75.4%) after a booster dose (Table 3). Among persons whose initial infection occurred between dose 2 and a booster dose, VE after the booster dose was 50.0% (95% CI = 26.9%–65.8%). Estimated VE of a booster dose was similar among persons aged <65 years (67.7%; 95% CI = 57.7%–75.3%) and ≥65 years (64.5%; 95% CI 56.0%–71.4%). Relative VE of a booster dose compared with ≥5 months after dose 2 was 55.9% (95% CI = 43.6%–65.5%).

**TABLE 3:** Estimated vaccine effectiveness against hospitalization with COVID-19 after previous SARS-CoV-2 infection* among persons with initial infection occurring before the first vaccine dose, and by age group —United States, June 2021–February 2022

Characteristic	No. of case-patients^†^ (N = 3,761)	No. of control-patients^† ^(N = 7,522)	VE (95% CI)^§^	
			Unadjusted	Adjusted
**Infection before dose 1**				
Unvaccinated (Ref)	2,304	3,581	—	—
Any mRNA vaccine, 1 dose^¶,^**	161	412	42.5 (30.2–52.7)	43.1 (30.7–53.2)
Any mRNA vaccine, 2 doses^¶,^**	960	2,356	39.1 (32.9–44.7)	41.7 (35.5–47.3)
Any mRNA vaccine, booster dose^¶,^**	183	777	67.6 (61.1–73.0)	70.3 (64.1–75.4)
**Age ≥65 yrs**				
Unvaccinated (Ref)	823	1,196	—	—
Any mRNA vaccine, 1 dose	72	163	35.3 (11.6–52.6)	35.7 (11.9–53.1)
Any mRNA vaccine, 2 doses	520	1,167	33.5 (23.0–42.6)	33.4 (22.4–42.9)
Any mRNA vaccine, booster dose	169	659	64.9 (56.6–71.6)	64.5 (56.0–71.4)
**Age <65 yrs**				
Unvaccinated (Ref)	1,480	2,375	—	—
Any mRNA vaccine, 1 dose	89	250	46.0 (29.6–58.6)	45.7 (28.9–58.5)
Any mRNA vaccine, 2 doses	518	1,329	40.3 (32.0–47.6)	41.9 (33.5–49.2)
Any mRNA vaccine, booster dose	90	383	66.1 (55.9–74.0)	67.7 (57.7–75.3)

**Abbreviations:** NAAT = nucleic acid amplification test; Ref = referent group; VE = vaccine effectiveness.

* Initial diagnosis was based on a previous positive SARS-CoV-2 NAAT or clinical diagnosis of COVID-19 >90 days before the date of the NAAT associated with subsequent hospitalization. COVID-19 diagnosis was defined as a clinical encounter with any of the following *International Classification of Diseases, Tenth Revision* diagnostic codes: U07.1, J12.81, or J12.82.

^†^ Case-patients had a positive NAAT performed between 10 days before and 3 days after the date of hospital admission with a diagnosis of COVID-19-like illness; control-patients had a negative NAAT result. COVID-19–like illness diagnoses were defined based on others’ methods (https://www.nejm.org/doi/full/10.1056/nejmoa2110362, Supplement Table S2) and included acute respiratory illness (e.g., COVID-19, respiratory failure, or pneumonia) or related signs or symptoms (e.g., cough, fever, dyspnea, vomiting, or diarrhea) using diagnostic codes from the *International Classification of Diseases, Tenth Revision*. Patients were eligible for inclusion if the hospitalization-associated SARS-CoV-2 NAAT was performed during June 20, 2021 and February 24, 2022.

^§^ VE was calculated as [1 − odds ratio] x 100, estimated using conditional logistic regression in a test-negative design after matching by 2-week calendar period of NAAT associated with hospital admission, 10-year age group, and state of residence. Adjusted estimates accounted in addition for measured differences in sex, race/ethnicity (White non-Hispanic race: yes/no and Hispanic ethnicity: yes/no), number of clinical encounters during 2019 (0, 1–9, or ≥10), number of underlying conditions (0, 1, or >1), and days since previous infection (as a continuous variable). Underlying conditions were extracted from EHR clinical encounter data and classified based on a CDC list of conditions associated with the highest risk for COVID-19 (https://www.cdc.gov/coronavirus/2019-ncov/hcp/clinical-care/underlyingconditions.html, accessed March 23, 2022), including the following diagnoses: alcoholic liver disease, autoimmune hepatitis, bronchiectasis, bronchopulmonary dysplasia, cancer, cardiomyopathy, cerebrovascular disease, chronic kidney disease, cirrhosis, chronic obstructive pulmonary disease, coronary artery disease, current smoker, administration or prescription of nontopical glucocorticoids within the previous 12 months, heart failure, HIV, immune deficiency, administration or prescription of immunosuppressive medications within the previous 12 months, interstitial lung disease, nonalcoholic fatty liver disease, obesity, pulmonary arterial hypertension, pulmonary embolus, pregnancy, solid organ transplant, tuberculosis, and type 1 or 2 diabetes.

^¶^ Patients were categorized on the date of NAAT associated with hospitalization as unvaccinated if no COVID-19 vaccine had been received; after dose 1 if ≥14 days had elapsed since receipt of the first dose of an mRNA COVID-19 vaccine and before any second dose; after dose 2 if ≥14 days had elapsed since receipt of the second dose of an mRNA COVID-19 vaccine, and no subsequent dose was received; and after a booster dose if ≥14 days had elapsed since receipt of an mRNA booster dose administered ≥5 months after a second dose. Patients were excluded from the analysis if they received a non-mRNA COVID-19 vaccine; the day of the NAAT-associated hospitalization was <14 days after dose 1, dose 2, or a booster dose; dose 2 was received <14 days after dose 1; any booster dose was <5 months after dose 2, they received >3 doses of vaccine, or their previous positive NAAT result or COVID-19 diagnosis was after the most recent vaccine dose. VE was calculated using the unvaccinated group as the referent.

** Among persons with a previous infection <180 days and ≥180 days before dose 1, adjusted VE after dose 1 was 43.2% (95% CI = 25.3%–56.8%) and 36.8% (95% CI = 14.0%–53.5%), respectively; adjusted VE after dose 2 was 37.6% (95% CI = 29.6%–44.6%) for persons with a previous infection <180 days before dose 1 and 38.9% (95% CI = 28.2%–48.1%) for persons with a previous infection ≥180 days before dose 1; adjusted VE after a booster dose was 72.5% (95% CI = 65.2%–78.2%) for persons with a previous infection <180 days before dose 1 and 46.7% (95% CI = 24.9%–62.2%) for persons with a previous infection ≥180 days before dose 1.

## Discussion

Among persons with previous SARS-CoV-2 infection or COVID-19 diagnosis, receipt of a COVID-19 mRNA vaccine provided protection against subsequent COVID-19 hospitalization. The highest level of protection was conferred by a booster vaccine dose, with similar VE during the Delta- and Omicron-predominant periods (approximately 60%–70%). In contrast, VE of 1 or 2 doses declined from 50%–60% during the Delta-predominant to approximately 35% during the Omicron-predominant period. Receiving a booster dose conferred protection even if the previous infection occurred after receipt of the second vaccine dose. Findings from this report indicate that SARS-CoV-2 reinfections leading to COVID-19–associated hospitalizations are preventable by COVID-19 vaccination.

Benefit of vaccination after previous SARS-CoV-2 infection was also indicated by an analysis of surveillance data from New York City that estimated approximately 50%–70% protection against hospitalization from reinfection (*5*). A case-control analysis using surveillance data from Brazil estimated 90% protection by 2 doses of Pfizer-BioNTech vaccine against hospitalization or death after reinfection (*6*); the high estimated VE might partly reflect recent vaccination in the context of potential decreased infection-induced immunity. The similar estimated benefit from 1 or 2 vaccine doses in preventing reinfection leading to hospitalization in the current study is consistent with evidence that vaccination elicits a more rapid immunologic response if preceded by a SARS-CoV-2 infection^¶¶¶^ (*7*). In the current analysis, a booster dose offered superior protection against reinfection leading to hospitalization.

Immunity from previous SARS-CoV-2 infection wanes over time (*1*,*8*) and was lower against the Omicron variant compared with immunity against other virus variants (*2*). However, protection is estimated to have remained stable against SARS-CoV-2 reinfection leading to hospitalization or death (*2*). Previous studies have indicated that, in general, protection by a hybrid of infection-induced and vaccination-induced immunity is superior to that from either alone and is less likely to wane over time (*1*,*8*). Compared with unvaccinated persons without previous infection, persons with a booster dose of mRNA vaccine have been estimated to have 90% protection against hospitalization with COVID-19 during the Omicron period; the highest estimated protection was among vaccinated persons with previous infection.****

The findings in this report are subject to at least five limitations. First, underascertainment of vaccination status from available information would likely lead to an underestimation of VE, particularly if vaccinated control-patients were misclassified as unvaccinated; this might have led to lower estimated VE compared with similar analyses (*5*,*6*,*9*). Second, generalizability might be limited by incomplete data or by missing data from persons who do not seek health care; however, Cosmos data are broadly representative of the U.S. population (*4*). Third, several VE estimates were imprecise, with broad CIs; estimates should be interpreted with caution. Fourth, underascertainment of previous infection might have occurred because of dependence on EHR data; however, findings were similar when restricting analyses to case-patients with positive initial NAAT results, and the test-negative design for an endpoint of severe illness mitigates the risk for selection bias. Finally, there might be residual or unmeasured confounding by characteristics associated with exposure, vaccination, or hospitalization that were not recorded in the data set.

An increasing proportion of the U.S. population has had SARS-CoV-2 infection^††††^ and might be at risk for SARS-CoV-2 reinfection leading to hospitalization. In the current analysis, approximately 50% of these reinfections occurred during the Omicron-predominant period. Vaccination remains the safest strategy for preventing complications of SARS-CoV-2 infection. COVID-19 vaccination offers additional protection against reinfection leading to hospitalization, with a booster dose offering the highest level of protection. To prevent COVID-19–associated hospitalization, all eligible persons should stay up to date with vaccination, including those with previous SARS-CoV-2 infection.

**Summary**

**What is already known about this topic?**

Persons with previous SARS-CoV-2 infection have some protection against reinfection leading to hospitalization, but there is limited evidence regarding the additional benefit of vaccination among these persons.

**What is added by this report?**

Among persons with previous infection, COVID-19 mRNA vaccination provided protection against subsequent COVID-19–associated hospitalization. Estimated vaccine effectiveness against reinfection leading to hospitalization during the Omicron-predominant period was approximately 35% after dose 2, and 68% after a booster dose.

**What are the implications for public health practice?**

To prevent COVID-19–associated hospitalization, all eligible persons should stay up to date with vaccination, including those with previous SARS-CoV-2 infection.

## Footnotes

* These authors contributed equally to this report.

† A test-negative design is a type of vaccine effectiveness study that compares the vaccination status of persons who seek testing in the same way (in this study, with COVID-19–like illness) and received either positive results (case-patients) or negative results (control-patients). Potential selection bias is limited by including patients who receive positive or negative test results but are otherwise similar. Vaccine effectiveness is estimated as the percentage of protection by being in a specified vaccination group compared with a referent group.

§ Cosmos is an aggregated EHR data platform of participating health systems that use software provided by Epic Systems Corporation that includes more than 135 million patients with similar demographics to U.S. Census data. Of 154 health systems included in the Cosmos dataset, 130 health systems that had data available since 2019 were included in the current analysis. https://epicresearch.org/about-cosmos

¶ COVID-19–like illness diagnoses included acute respiratory illness (e.g., COVID-19, respiratory failure, or pneumonia) or related signs or symptoms (cough, fever, dyspnea, vomiting, or diarrhea) using diagnosis codes from the *International Classification of Diseases, Tenth Revision*.

** COVID-19 was defined as a clinical encounter with any of the following *International Classification of Diseases, Tenth Revision* diagnostic codes: U07.1, J12.81, and J12.82. A difference of >90 days was used consistent with the Council of State and Territorial Epidemiologists case definition of COVID-19: https://ndc.services.cdc.gov/case-definitions/coronavirus-disease-2019-2021/.

†† Patients were categorized on the date of NAAT associated with hospitalization as unvaccinated if no COVID-19 vaccine had been received; after dose 1 if ≥14 days had elapsed since receipt of the first dose of an mRNA COVID-19 vaccine and before any second dose; and after dose 2 if ≥14 days had elapsed since completion of a second mRNA vaccine dose, and no subsequent dose was received. To limit early additional doses (for example, among immunocompromised persons), patients were categorized as after booster if ≥14 days had elapsed since receipt of an mRNA booster dose administered ≥5 months after a second dose, and if no further doses had been received. Patients were excluded from the analysis if they received a non-mRNA COVID-19 vaccine; the day of the NAAT-associated hospitalization was <14 days after dose 1, dose 2 or a booster dose; dose 2 was received <14 days after dose 1; any booster dose was <5 months after dose 2; they received >3 doses of vaccine; or their previous positive NAAT or COVID-19 diagnosis was after the most recent vaccine dose. In addition, if patients had more than one hospitalization-associated NAAT, they were considered a case-patient if their NAAT was positive at any point and were excluded from being a control-patient, (i.e., cases-patients could not also serve as control-patients).

§§ Vaccination information for each patient was collected from four sources: 1) vaccine doses administered within the health system, 2) electronic health records, 3) vaccination information obtained from other health systems using a shared data platform, and 4) vaccination information obtained directly from a state or other vaccine registry.

¶¶ Characteristics were classified on the date of the NAAT associated with the hospital admission. Underlying conditions were extracted from EHR clinical encounter data and were based on a CDC list of conditions associated with the highest risk for COVID-19 (https://www.cdc.gov/coronavirus/2019-ncov/hcp/clinical-care/underlyingconditions.html, accessed March 23, 2022) and included the following: alcoholic liver disease, autoimmune hepatitis, bronchiectasis, bronchopulmonary dysplasia, cancer, cardiomyopathy, cerebrovascular disease, chronic kidney disease, cirrhosis, chronic obstructive pulmonary disease, coronary artery disease, current smoker, receipt of nontopical glucocorticoids within the previous 12 months, heart failure, HIV, immune deficiency, receipt of immunosuppressive medications within the previous 12 months, interstitial lung disease, nonalcoholic fatty liver disease, obesity, pulmonary arterial hypertension, pulmonary embolus, pregnancy, solid organ transplant, tuberculosis, and type 1 or 2 diabetes.

***
https://covid.cdc.gov/covid-data-tracker/#variant-proportions

††† 45 C.F.R. part 46, 21 C.F.R. part 56; 42 U.S.C. Sect. 241(d); 5 U.S.C. Sect. 552a; 44 U.S.C. Sect. 3501 et seq.

§§§ Among 25,641 patients with a positive NAAT >90 days after an initial positive SARS-CoV-2 NAAT or diagnosis of COVID-19 during June 20–December 31, 2021, 2,378 (9.3%) were admitted to a hospital with COVID-19–like illness between 10 days after and 3 days before the subsequent positive NAAT result, indicating reinfection with COVID-19–associated hospitalization.

¶¶¶ https://www.medrxiv.org/content/10.1101/2021.12.23.21268285v1

****
https://www.medrxiv.org/content/10.1101/2022.03.22.22272745v1

††††
https://covid.cdc.gov/covid-data-tracker/#antibody-seroprevalence
